# An Intraoperative Trajectory‐Determined Strategy of Patient‐Specific Drill Template for C_2_ Transoral Pedicle Insertion in Incomplete Reduction of Atlantoaxial Dislocation: An *In Vitro* Study

**DOI:** 10.1111/os.13049

**Published:** 2021-06-06

**Authors:** Jing Shan, Mei‐song Zhu, Lu‐tao Li, Peng Peng, Min Dai, Li‐jun Lin, Jian‐yi Li

**Affiliations:** ^1^ Department of Orthopaedics, Artificial Joints Engineering and Technology Research Center of Jiangxi Province The First Affiliated Hospital of Nanchang University Nanchang China; ^2^ Department of Joint and Orthopaedics, Zhujiang Hospital Southern Medical University Guangzhou China; ^3^ Department of Orthopaedics TCM‐Integrated Hospital of Southern Medical University Guangzhou China; ^4^ Department of Anatomy, Guangdong Provincial Key Laboratory of Medical Biomechanics, School of Basic Medical Sciences Southern Medical University Guangzhou China; ^5^ Nanhai Hospital Southern Medical University Foshan China

**Keywords:** Atlantoaxial joint, Pedicle screw, 3D printing, Cadaver, Classification

## Abstract

**Objectives:**

This study aims to explore a novel intraoperative trajectory‐determined strategy of grouped patient‐specific drill templates (PDTs) for transoral C_2_ pedicle screw insertion (C_2_TOPI) for atlantoaxial dislocation (AAD) with incomplete reduction and to evaluate its efficiency and accuracy.

**Methods:**

Ten cadaveric C_2_ specimens were scanned by computed tomography (CT) and randomly divided into two groups (the PDT and freehand groups). A novel intraoperative trajectory‐determined strategy of grouped PDTs was created for AAD with incomplete reduction. C_2_TOPI was performed by use of the PDT technique and the fluoroscopy‐guided freehand technique. After surgery, the screw deviations from the centroid of the cross‐section at the midpoint of the pedicle and screw position grades were assessed in both groups.

**Results:**

Compared to the freehand group, the PDT group had a significantly shorter surgery time than the freehand group (47.7 *vs* 61.9 min, *P* < 0.001). The absolute deviations from the centroids between the preoperative designs and postoperative measurements on the axial plane of the pedicle were 1.19 ± 0.25 mm in the PDT group and 1.82 ± 0.51 mm in the freehand group. On the sagittal plane of the pedicle, the corresponding values were 1.10 ± 0.33 mm in the PDT group and 1.70 ± 0.49 mm in the freehand group. The absolute deviations of the free‐hand group on both the axial and sagittal planes were higher than that of the freehand group (*P <* 0.05 and *P <* 0.05, respectively). For the grade of screw insertion position, nine (90%) were observed in type I and one (10%) in type II in the PDT group, whereas five (50%) were in type I, three (30%) were in type II, and two (20%) in type III in the freehand group. Statistical differences could not be found between the groups in terms of the screw positions (*P* > 0.05).

**Conclusion:**

The novel intraoperative trajectory‐determined strategy of grouped PDTs can be used as an accurate and feasible method for C_2_TOPI for AAD with incomplete reduction.

## Graphical abstract

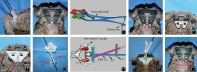

An intraoperative trajectory‐determined strategy of grouped patient‐specific drill templates (PDTs) was developed for atlantoaxial dislocation (AAD) with incomplete reductions. Their efficacies and accuracies in transoral C_2_ pedicle insertion (C_2_TOPI) were further evaluated. We found the intraoperative trajectory‐determined PDTs could effectively facilitate C_2_TOPI in AAD with incomplete reductions.

## Introduction

Atlantoaxial dislocation (AAD) can cause neck pain, limitations in neck movement, or spinal cord compression. Thus, surgical intervention is usually needed to relieve spinal compression and restore stability between the atlas and the axis in patients^1^, ^2^. In recent years, a specially designed reduction, described as transoral atlantoaxial reduction plate (TARP) surgery, has been widely confirmed to be an advanced treatment for AAD^1^, ^3^, ^4^, ^5^ that could allow transoral release, reduction, internal fixation, and bone grafting in a one‐stage operation. Transoral C_2_ pedicle fixation was introduced in the third generation of the TARP system^6^, which could effectively provide better biomechanical performance than intravertebral insertion fixation. Generally, transoral C_2_ pedicle screw insertion (C_2_TOPI) is considered risky because it is proximal to the vital structures such as the vertebral arteries, the spinal cord, and the nerve roots^7^. Therefore, accurate C_2_TOPI is the key to successful clinical application of the TARP system.

Various strategies have been developed for safe and accurate screw insertion in C_2_TOPI. Anatomical free‐hand technique was routinely used in clinical practice; however, high perforation incidences from 15% to 17.3% were reported^8^, ^9^, ^10^. C‐arm fluoroscopy which could provide detailed information of the spinal anatomy was thus introduced to facilitate C_2_TOPI^11^, ^12^. However, the overlapping images are not sufficient for atlantoaxial complexity and therefore cannot indicate the screw position accurately^13^. Li *et al*.^14^ found that the medial and lateral cortical breach rate of C_2_TOPI was as high as 46.9% performed by the fluoroscopic‐guided freehand technique. The recent development of intraoperative three‐dimensional (3D) fluoroscopy‐based or computed tomograghy (CT)‐based navigation could provide accurate 3D images of the spinal anatomy that could be used for precise pedicle screw placement^13^, ^15^, ^16^. However, these devices are relatively expensive, as well as the operative procedures being complicated and time‐consuming, which influences their wide application in clinical trials^13^, ^17^. Patient specific drill templates (PDTs) produced by 3D printing were then introduced to assist C_2_TOPI, which have been confirmed to improve the accuracy and efficacy of C_2_TOPI as well as obviate complex equipment and time‐consuming procedures^18^, ^19^. However, these strategies used for PDTs were mainly based on the pre‐set trajectory, which could not meet the demands for all kinds of intraoperative reduction in AAD.

Actually, there are two cases of intraoperative reduction of AAD—complete and incomplete reductions^1^, ^4^—which require different strategies for PDT design. For AAD with complete reduction, surgeons need the optimal trajectories of C_2_TOPI, and the aforementioned traditional PDT strategy that determines the trajectories preoperatively might fulfill these demands. However, for AAD with incomplete reduction, because the C_2_TOPI trajectories would be determined after the intraoperative reduction, the traditional PDT approach may not meet the demands. Thus, a novel PDT approach remains to be developed. Therefore, in this study, we aimed to (i) develop a novel intraoperative trajectory‐determined strategy of grouped PDTs specifically for incomplete reduction of AAD and evaluate the (ii) efficiency and (iii) accuracy of this technique in facilitating C_2_TOPI. We hypothesized that this grouped PDT might be an alternative to the fluoroscopy‐guided freehand technique for C_2_TOPI.

## Materials and Methods

### Specimens

A total of 10 formalin‐preserved cadaveric cervical spines from 10 cadavers (five males and five females aged from 50 to 67 years, with a mean age of 61 years) were harvested and scanned by CT (Philips, Eindhoven, The Netherlands). All cervical vertebrae that fulfilled the following inclusion criteria were included in the study: no bone defects or fractures according to the CT scan images. The CT data were saved in the DICOM (Digital Imaging and Communications in Medicine) format at 0.625 mm intervals with a pixel size of 0.55 mm. Twenty sides of the cadaveric cervical spines were then randomized to undergo C_2_TOPI surgeries through either the PDT technique or the fluoroscopy‐guided freehand technique. The PDT design and accuracy assessment were performed by a postgraduate student; the C_2_TOPI surgeries were performed by another attending spinal surgeon. Neither knew whether the specimens belonged to the PDT or freehand group. All specimens underwent internal atlantoaxial fixations with the fourth generation of the TARP system, which added vertebral body screws (VBSs) for fixation based on the third generation of the TARP system^1^.

### Determination of the Centroid at the C_2_ Pedicle

**Fig. 1:**
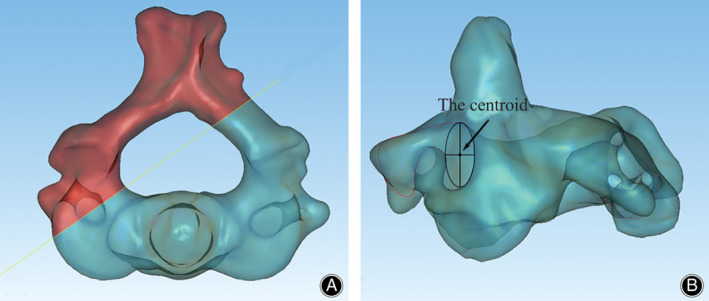
Calculation of the centroid of the cross‐section at the midpoint of the pedicle of C_2_. (A) The midpoint and corresponding cross‐section of each pedicle were determined. (B) After the contour of the cross section was fitted by a maximum ellipse, the centroid was calculated from the maximum ellipse (reprinted from our previous research^18^ with permission from Copyright Clearance Center).

All specimens were handled by the same procedures in our previously published protocols^18^. The CT data were imported into Mimics software version 14.11 (Materialize Corp., Leuven, Belgium) to obtain 3D reconstruction models of the C_1_ and C_2_ vertebrae. The 3D model of C_2_ was then exported to Geomagic Studio software version 11.0 (3D Systems Corp, Morrisville, NC) in STL format. The midpoint and corresponding cross section of each pedicle were determined using this software. After the contour of the cross section was fitted by a maximum ellipse, the centroid of the cross section at the midpoint of the pedicle was subsequently calculated from the maximum ellipse (Fig. 1).

### Design and Manufacture of Grouped PDTs for AAD with Incomplete Reduction

**Fig. 2:**
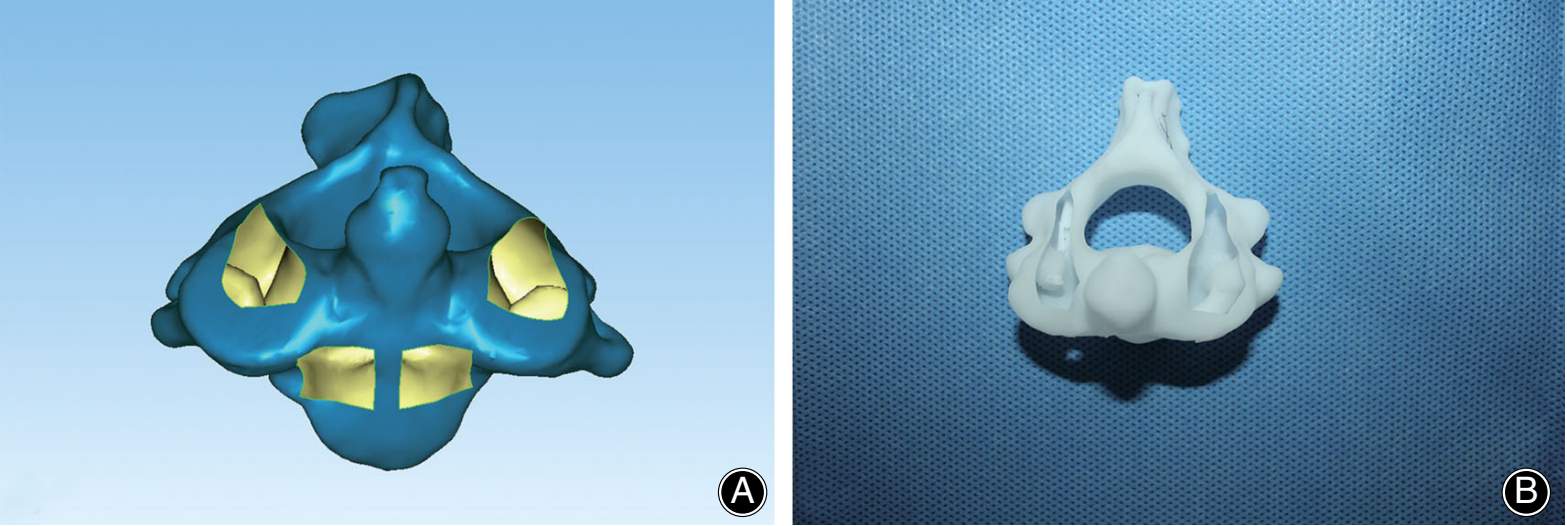
3D model of the C_2_ vertebra. (A) 3D C_2_ model in which the surfaces of the upper pedicle and anterior vertebra body were removed. (B) 3D‐printed model.

A novel intraoperative trajectory‐determined strategy of grouped PDTs for C2TOPI was developed for AAD with incomplete reduction. The C_2_ 3D models were further handled through Geomagic Studio 11.0 software, in which the upper surface of the pedicle and the anterior surface of the vertebral body were removed. Then, the C_2_ shell model was extracted and subsequently 3D printed by a RS6000 stereolithography printer (Shanghai Union Technology Corp, Shanghai, China). The structures of the 3D‐printed C_2_ models exposed the inner wall of the vertebral artery and the lateral wall of the vertebral canal, which clearly showed the insertion position of the K‐wire in the pedicle when it was inserted from the anterior aspect of C_2_ (Fig. 2).

**Fig. 3:**
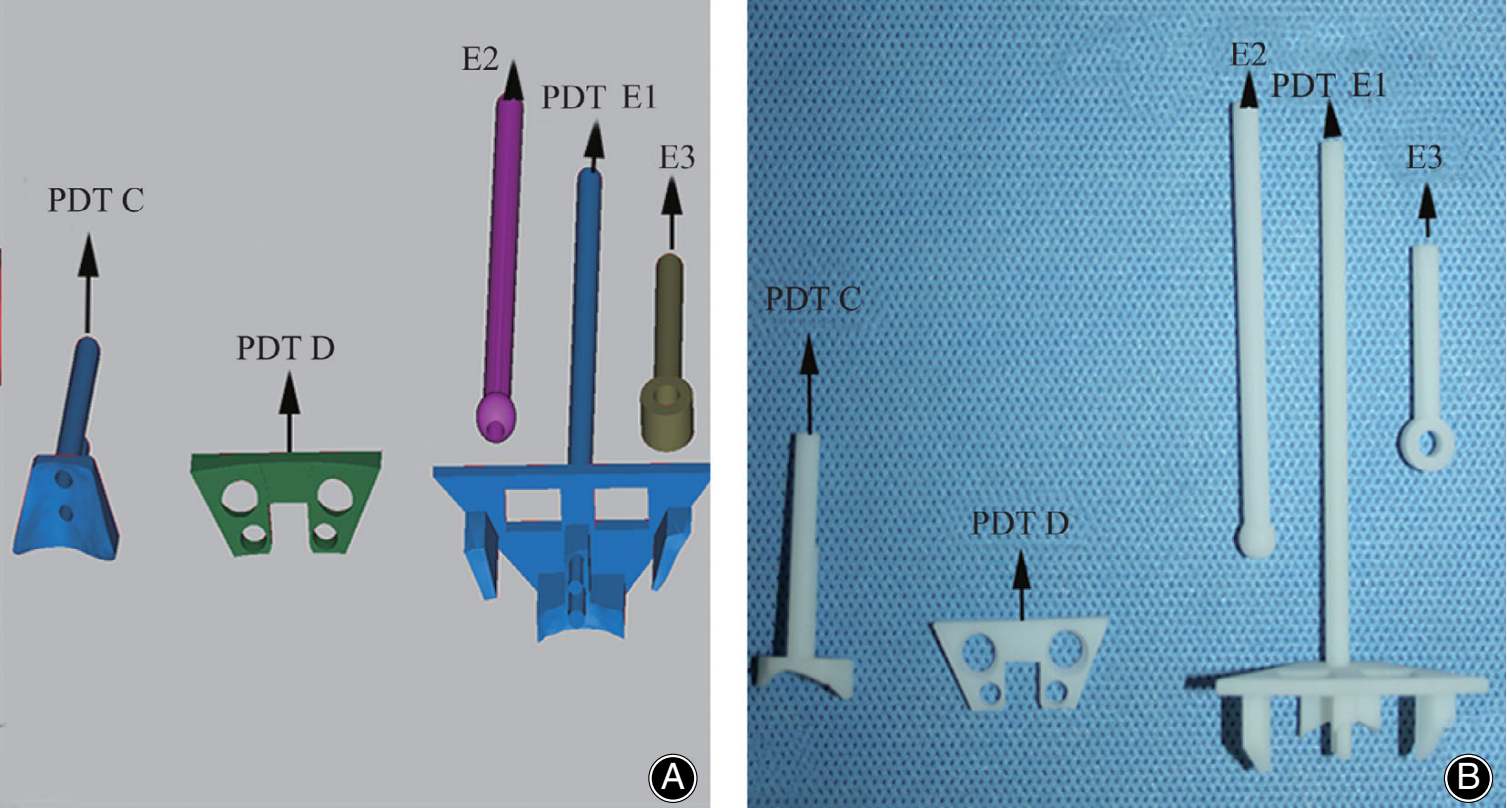
Grouped PDTs for C_2_TOPI. (A) The design models of PDT C, D, and E. (B) 3D‐printed models of PDT C, D, and E.

The 3D models of C_1_, C_2_, and the TARP were also imported into SolidWorks 2014 (Dassault Systemes, France), in which the PDTs were designed. There were three PDT parts for C_2_TOPI: PDT C, D, and E (Fig. 3A). PDT C was used to guide the holes for temporary reduction screw (TRS) and setscrew, and had a bottom surface and two guide tubes. The bottom surface was the reverse surface of the anterior surface of the vertebral body, by which PDT C could tightly attach to C_2_. The two guide tubes were perpendicular to the bottom surface, and the guide tube that was used to guide for the setscrew was 5 mm higher than that for the TRS. PDT D was designed to fit the TARP surface and was used to locate the intraoperative position of the TARP after atlantoaxial reduction. The inferior two holes were positioned in accordance with the inferior two holes of the TARP, which were used to screw in VBS. The upper two holes of PDT D were used to determine the entrances of the trajectories. PDT E was used for C_2_TOPI drilling. Since the most areas of the anterior surface of C_2_ were covered by the TARP in the operative procedure, a special “table” structure was designed. The bottom surfaces of the columns under the table and two short pins were aligned to the TARP‐uncovered surface of C_2_ and two holes for a TRS and setscrew. On the table, three removable components (E1, E2, and E3) were designed to guide the K‐wire insertion when they were immobilized. These PDTs were created using the same 3D‐printing technique described above (Fig. 3B).

### Cadaver for C_2_TOPI

**Fig. 4:**
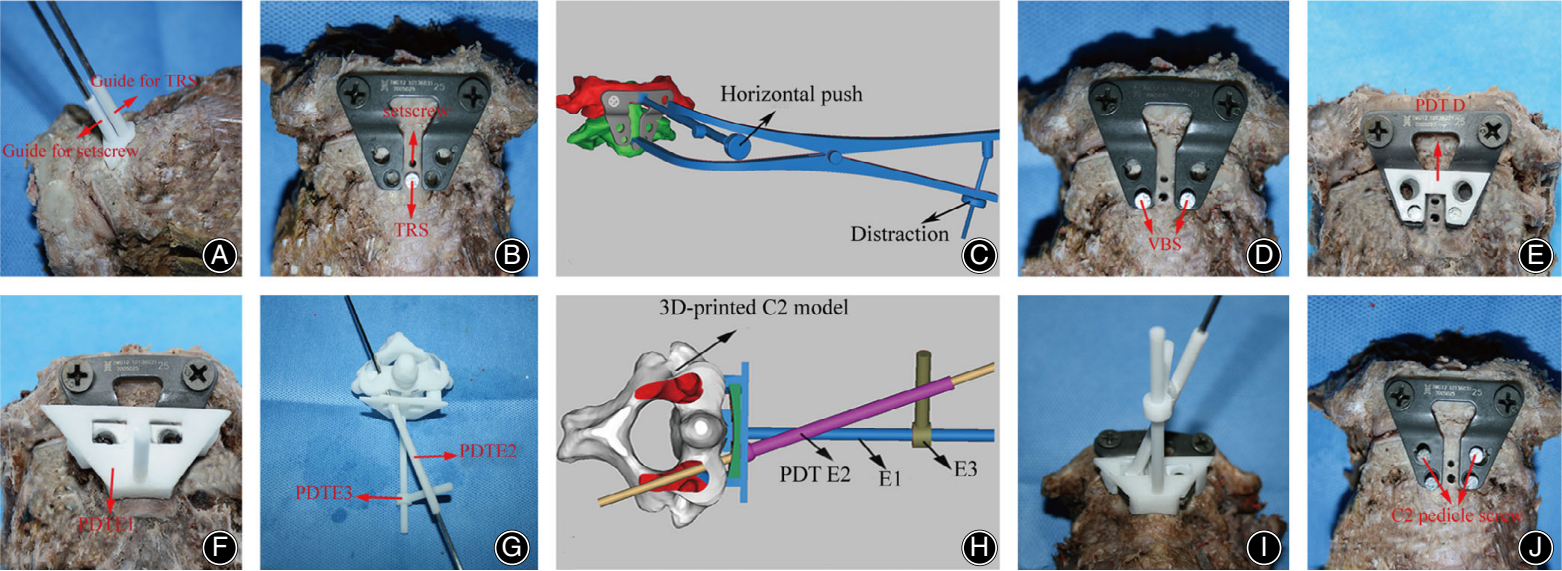
Cadaveric C_2_TOPI with the assistance of PDT C, D, and E. (A) PDT C was used to guide the insertions of the TRS and setscrew. (B) A TRS was then screwed in the lower hole, and the upper two holes of the TARP were fixed to C_1_ with lateral mass screws. (C) After the TRS was screwed in, C_1_ and C_2_ were relocated to a proper position with the reduction instrument because it might not obtain the complete reduction (surgical simulation diagrams). (D) The TARP was then temporarily fixed with VBSs. (E) PDT D was used to find the intraoperative position of the TARP according to the positions of the VBSs. The entrance points of C2TOPI were determined. (F) After removing the TRS, PDT E1 was attached to the anterior surface of C_2_, and the relative position between PDT D and E1 was agglutinated with a medical glue. (G) PDT D and E1 were transferred to the 3D‐printed C_2_ model to determine the drilling direction through visual observation. A K‐wire was inserted into the pedicle through the drilling tube (PDT E2). The position of the K‐wire was adjusted through visual observation to locate it at the central C_2_ pedicle. The removable components of PDT E1, E2, and E3 were agglutinated together using the same medical glue to determine the drill direction. (H) Simulation diagrams which showed PDT E1, E2, and E3 were agglutinated to determine the drilling direction. (I) PDT D and E were switched back to the cervical specimen to guide C_2_TOPI with the determined entrance point and drilling direction. (J) C_2_TOPI was completed after the two 3.5‐mm diameter screws were inserted.

The 10 cadavers were operated on by an attending spinal surgeon with either the PDT‐guided or freehand technique in random order. The anterior surfaces of C_1_ and C_2_ were exposed and the soft tissues were removed until the bony surface could be clearly observed. Then the transverse ligament and other soft tissues around the odontoid were resected to create simulated AADs between C_1_ and C_2_. In the PDT group, PDT C was placed on the anterior surface of C_2_ until a lock‐and‐key configuration was achieved. K‐Wires were then inserted to drill the holes for TRS and setscrew through the two guide tubes of PDT C (Fig. 4A). A TRS was then screwed in the lower hole, and the upper two holes of the TARP were fixed to C_1_ with lateral mass screws (Fig. 4B). After the TRS was screwed in, C_1_ and C_2_ were relocated to a proper position with the reduction instrument because it might not obtain the complete reduction (Fig. 4C). VBSs were then screwed in for temporary fixation of the TARP (Fig. 4D). PDT D was used to find the intraoperative TARP position according to the positions of the VBSs. Thus, the C2TOPI entrance points were determined (Fig. 4E). After removing the TRS, PDT E1 was then assembled to attach to the anterior surface of C_2_ that was not covered by the TARP. The relative position between PDT D and E1 was agglutinated with a medical glue (COMPONT®, Beijing Compont Medical Devices Co., Ltd., Beijing, China), which is a synthetic cyanoacrylic glue that contains the n‐butyl‐2‐cyanoacrylate formulation and is approved by the China Food and Drug Administration (CFDA) as a Class III medical device (Fig. 4F). PDT D and E1 were transferred to the 3D‐printed C_2_ model to determine the drilling direction. A K‐wire was inserted into the pedicle through the drilling tube (PDT E2). The position of the K‐wire was adjusted through visual observation to locate it at the central C_2_ pedicle. The removable components of PDT E1, E2, and E3 were then agglutinated together using the same medical glue (COMPONT®) to determine the drill direction (Fig. 4G,H). PDT D and E were switched back to the cervical specimen to guide C_2_TOPI with the determined entrance point and drill direction (Fig. 4I). C2TOPI was completed after the two 3.5‐mm diameter screws (Kangli Orthopedic Instrument, Jiangsu, China) were inserted (Fig. 4J). In the fluoroscopy‐guided freehand group, the same attending surgeon performed the C_2_TOPI surgeries, applying the technique described in previous publications^1^, ^14^.

### Comparison of the Operation Time and Screw Placement Accuracy Between the Two Groups

The operation time for C_2_TOPI surgeries of both groups were recorded. After C2TOPI surgeries, a postoperative CT scan was performed, and 3D reconstructions of the cadaveric C_2_ vertebrae with the inserted screws were created with the same procedures. The screw insertion accuracy assessment was performed using Geomagic Studio 11.0 software. The postoperative centroids of the inserted screw at the midpoint of the pedicle in both the PDT and freehand groups were extracted. There were two assessment standards used to evaluate the insertion accuracy: one of the standards was the deviations of the centroids on the axial and sagittal planes of the pedicle between the preoperative design and postoperative screw position. The axial plane deviations towards the lateral side were recorded as positive values, and the deviations towards the medial side were recorded as negative values. The sagittal plane deviations towards the superior and inferior sides were recorded as positive and negative values, respectively. Another intuitive assessment criterion was the grade of the pedicle screw insertion position, which were graded according to the modified All India Institute of Medical Sciences outcome‐based classification^18^:

Type I: ideal placement—screw threads are completely within the bony cortex.

Type II: acceptable placement—less than 50% of the diameter of the screw violates the surrounding cortex.

Type III: unacceptable placement—clear violation of the transverse foramen or spinal canal.

### Statistical Analysis

The results including the surgery time and deviations are presented as the means ± standard deviations. Independent samples *t*‐tests were used to analyze the absolute values of the deviations between the two groups on the axial and sagittal planes as well as the surgery time. The chi‐square test was performed to compare the pedicle screw position between the two groups. SPSS 20.0 software (IBM Corporation, Armonk, NY, USA) was used for all analyses, and the significance was defined as *P* < 0.05.

## Results

### Comparison of the Operation Time Between the PDT and Freehand Groups

All PDTs were produced successfully using 3D reconstruction and 3D printing. During the operation, the PDTs were fitted to their corresponding anterior cervical surfaces appropriately without any free movement. K‐Wires were easily inserted into the cervical pedicle with the assistance of the PDTs. The surgery time was 47.7 ± 4.49 min in the PDT group and 61.9 ± 3.21 min in the freehand group. Compared to the freehand group, the PDT group had a significantly shorter surgery time than the freehand group (*t* = 8.1, *P* < 0.001).

### Evaluation of the Absolute Deviations from the Centroids Between the PDT and Freehand Groups

The absolute deviations from the centroids between the preoperative designs and postoperative measurements on the axial plane of the pedicle were 1.19 ± 0.25 mm in the PDT group and 1.82 ± 0.51 mm in the freehand group. On the sagittal plane of the pedicle, the corresponding values were 1.10 ± 0.33 mm in the PDT group and 1.70 ± 0.49 mm in the freehand group (Table 1). The absolute deviations of the free‐hand group on both the axial and sagittal planes were higher than that of the freehand group (*t* = −3.42, *P* = 0.003 and *t* = −3.163, *P* = 0.006, respectively).

**TABLE 1:** The absolute deviations from the centroids between the preoperative designs and postoperative measurements on the PDT and freehand groups

		PDT group	Freehand group	*t*	*P*
Absolute deviations on the axial plane (mm)		1.19 ± 0.25	1.82 ± 0.51	3.42	0.003
Absolute deviations on the sagittal plane (mm)		1.10 ± 0.33	1.70 ± 0.49	3.163	0.006

### Evaluation of the Pedicle Screw Insertion Position Between the PDT and Freehand Groups

For the grade of screw insertion position, there are nine (90%) in type I and one (10%) in type II in the PDT group, whereas there are five (50%) in type I, three (30%) in type II, and two (20%) in type III in the freehand group (Table 2). There were no significant differences between the two groups (*χ* ^2^ = 3.651, *P* = 0.173).

**TABLE 2:** The grade of screw insertion position between the PDT and freehand groups

	Groups			
Grade of screw position	PDT group	Freehand group	*χ* ^2^	*P*
Type I	9 (90%)	5 (50%)	3.651	0.173
Type II	1 (10%)	3 (30%)		
Type III	0	2 (20%)		

## Discussion

It is well‐known that intraoperative reduction of AAD can be divided into complete and incomplete reductions, which require different strategies for PDT design in assisting C_2_TOPI. For AAD with complete reduction, Li *et al*.^19^ developed grouped PDTs in their cadaveric study, in which several graded screw trajectories were pre‐set to facilitate C_2_TOPI. However, the operation of these PDTs in assisting C_2_TOPI were complex. Therefore, we simplified the design strategy of PDTs and developed an easy‐to‐apply grouping of preoperative‐trajectory‐determined PDTs in our previous study^18^. These PDTs have been proved to provide surgeons with an accurate and easy‐to‐apply method to facilitate C_2_TOPI. However, for AAD with incomplete reduction, to our best knowledge, there has been no report on C_2_TOPI using 3D‐printed templates. Therefore, in the present study, we firstly developed a novel intraoperative trajectory‐determined strategy of grouped PDTs specifically for C_2_TOPI in AAD with incomplete reduction, and the efficiency and veracity were validated and compared with those of the fluoroscopy‐guided freehand technique.

### PDTs for C_2_TOPI for AAD with Incomplete Reduction

For AAD with complete reduction, the ideal trajectory of C_2_TOPI that is considered to not only take into account screw insertion safety but also biomechanical properties could be preoperatively designed for this procedure. But in this study, we primarily focused on achieving a safe trajectory of C_2_TOPI for AAD with incomplete anatomical reduction because the trajectories of C_2_TOPI can be determined only after intraoperative reduction. A PDT might be subsequently developed to facilitate C_2_TOPI. However, due to the coverage of the C_2_ anterior surface by the TARP after incomplete reduction of AAD, and the various relative positions among C_1_, C_2_, and the TARP, the usual structures of PDTs which determined the trajectories preoperatively and were created using an inverse massive bone surface were not feasible for AAD with incomplete reduction^20^, ^21^, ^22^. A novel intraoperative trajectory‐determined strategy of grouped PDTs for C_2_TOPI was developed. There were three steps in facilitating C_2_TOPI with the PDT. The first step was to facilitate an intraoperative reduction that allowed a TRS, setscrew, and PDT E to find the preoperatively designed positions. PDT C was designed for this step and allowed the determination of the two hole positions for the TRS and setscrew, which were also aligned to the two short pins of PDT E. The second step was to facilitate the location of the entry point of C_2_TOPI after the reduction, which is located at the centre of the pedicle screw holes of the TARP. The determined TARP, including the inferior two holes for VBSs and the central two holes for C_2_TOPI entrances, were used for the location registration. PDT D, which included the inferior two holes and the upper two holes, was specifically designed. PDT D was firmly attached to the anterior surface of the TARP, with good surface registration between the holes. The entry point of C_2_TOPI was then successfully determined. The last step was to facilitate the trajectory drilling of C_2_TOPI. PDT E was designed, which included a table, the beneath registration columns/pins and three above removable components (E1, E2, and E3). The table and the beneath registration columns/pins were the same as those of PDT B in our previous study^18^, and the three above removable components were used to obtain the drill direction of C2TOPI. When the K‐wire through the removable drilling tube was located at the central pedicle of the 3D‐printed C_2_ model, the removable components were then agglutinated together to determine the drilling direction. Then, the entry point and drilling direction of C_2_TOPI were locked by the combined PDTs to allow drilling with a safe trajectory.

### Efficiency and Accuracy of PDTs in Facilitating C_2_TOPI

To ensure the efficiency and accuracy of PDTs in facilitating C_2_TOPI, in the present study, we applied the same means that were used to evaluate the outcome of PDT for AAD with complete reduction^18^. To show the efficiency of PDTs, we calculated the surgery time of the two groups. The surgery time of the PDT group was much shorter than that of the freehand group when performed by the same surgeon, which revealed that the use of a PDT could improve the efficiency of surgeons to facilitate C_2_TOPI. The accuracy of PDTs in facilitating C_2_TOPI is also of vital importance, and two assessment standards were used to evaluate the insertion accuracy: one of the standards was the absolute value of the deviations from the centroids between the preoperative designs and postoperative measurements, which was used to show the actual deviations. Our study showed that the absolute deviations in the axial plane (1.19 ± 0.25 mm) and the sagittal plane (1.10 ± 0.33 mm) in the PDT group might be within an acceptable range for clinical application. These results were both significantly different from those in the freehand group. Another intuitive assessment criterion was the critical condition of the cortex being penetrated, which was evaluated by a rank index of the screw insertion position. In our study, no significant difference was observed in the screw positions between the two groups. However, two unacceptable breaches (type III) occurred in the freehand group. This rate was the same as in our previous report (20%)^18^ and slightly higher than that in previous reports by Li *et al*.^14^ (13.0%). This result most likely occurred because the surgeon was only an attending spinal surgeon with minimal experience in C_2_TOPI, and the sample size was relatively small. Generally, the results including the surgery time, quantitative absolute values of the deviations and intuitive rank revealed that the PDT‐guided technique was more efficient and precise than the fluoroscopy‐guided freehand technique in facilitating C_2_TOPI.

### Limitations

Although the novel PDT strategy has obvious advantages in facilitating C_2_TOPI, some limitations need more attention. First, these PDTs were mainly applied for simple atlantoaxial dislocation with relatively intact bony structure. For the patients with anterior C_2_ fracture, these PDTs cannot fit well to the fractured surface of C_2_, thus limiting its clinical application^23^. Second, the placement of the group PDTs and the reduction instruments were demonstrably difficult in terms of transoral accessibility in a deep surgical field, and the cadaveric simulation experiment did not fully represent the relevant intraoperative situation. Therefore, the aim of this study was only to introduce a novel technical strategy of the PDTs for AAD with incomplete reduction and to further evaluate the efficiency and accuracy of PDTs in facilitating C_2_TOPI. Further improvements can be made in clinical applications, depending on the actual situation.

### Conclusion

In summary, the novel intraoperative trajectory‐determined strategy of grouped PDTs could be used as an accurate and feasible method for C2TOPI for AAD with incomplete reduction. This could provide a viable alternative for surgeons.
